# UDP-Sugar Producing Pyrophosphorylases: Distinct and Essential Enzymes With Overlapping Substrate Specificities, Providing *de novo* Precursors for Glycosylation Reactions

**DOI:** 10.3389/fpls.2018.01822

**Published:** 2019-01-04

**Authors:** Daniel Decker, Leszek A. Kleczkowski

**Affiliations:** Department of Plant Physiology, Umeå Plant Science Centre, Umeå University, Umeå, Sweden

**Keywords:** carbohydrate biosynthesis, chemical genetics, nucleotide sugar synthesis, enzyme substrate specificity, UDP-glucose pyrophosphorylase, UDP-*N*-acetylglucosamine pyrophosphorylase, UDP-sugar pyrophosphorylase

## Abstract

Nucleotide sugars are the key precursors for all glycosylation reactions and are required both for oligo- and polysaccharides synthesis and protein and lipid glycosylation. Among all nucleotide sugars, UDP-sugars are the most important precursors for biomass production in nature (e.g., synthesis of cellulose, hemicellulose, and pectins for cell wall production). Several recent studies have already suggested a potential role for UDP-Glc in plant growth and development, and UDP-Glc has also been suggested as a signaling molecule, in addition to its precursor function. In this review, we will cover primary mechanisms of formation of UDP-sugars, by focusing on UDP-sugar metabolizing pyrophosphorylases. The pyrophosphorylases can be divided into three families: UDP-Glc pyrophosphorylase (UGPase), UDP-sugar pyrophosphorylase (USPase), and UDP-*N*-acetyl glucosamine pyrophosphorylase (UAGPase), which can be distinguished both by their amino acid sequences and by differences in substrate specificity. Substrate specificities of these enzymes are discussed, along with structure-function relationships, based on their crystal structures and homology modeling. Earlier studies with transgenic plants have revealed that each of the pyrophosphorylases is essential for plant survival, and their loss or a decrease in activity results in reproductive impairment. This constitutes a problem when studying exact *in vivo* roles of the enzymes using classical reverse genetics approaches. Thus, strategies involving the use of specific inhibitors (reverse chemical genetics) are also discussed. Further characterization of the properties/roles of pyrophosphorylases should address fundamental questions dealing with mechanisms and control of carbohydrate synthesis and may allow to identify targets for manipulation of biomass production in plants.

## Introduction

Many processes during a plant’s life are dependent on targeted-glycosylation of different compounds (carbohydrates, polysaccharides, lipids, proteins, hormones, etc.). These glycosylation are carried out by a group of enzymes called glycosyltransferases (GT), which catalyze the transfer of a sugar from an “activated” sugar, usually a nucleoside-diphosphate-sugar (NDP-sugar) to an acceptor (commonly *via* an oxygen but also *via* nitrogen, sulfur, or carbon moiety on the acceptor molecule) (Lairson et al., 2008). Besides the formation of NDP-sugars, some sugars can also be “activated” by linking with CMP (Kleczkowski and Decker, 2015), whereas certain oligosaccharides may link with dolichol pyrophosphate (Strasser, 2016). Plants contain several hundred genes coding for GTs, for example, 565 for *Arabidopsis thaliana* as of August 2017^1^. These GTs are separated into different classes, commonly GT-A and GT-B (but also GT-C) depending on the presence of distinct structural folds, and are also classified as inverting or retaining GTs, depending on whether the anomeric carbon of the sugar donor retains the same stereochemistry after bond formation to the acceptor (Coutinho et al., 2003; Lairson et al., 2008). Proper classification of the GTs based on their substrate specificities (for both NDP-sugar and acceptor) is currently ongoing.

**Figure 1:**
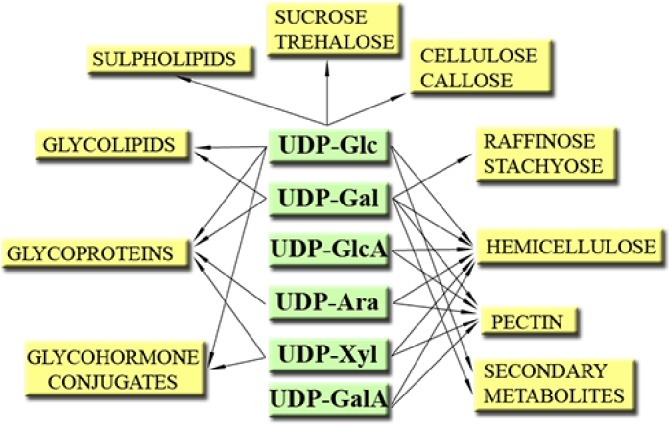
Some roles of UDP-sugars in plants. Modified from Kleczkowski et al. (2011a).

Plants form and utilize a number of important NDP-sugars, for example, GDP-based NDP-sugars such as GDP-Mannose (GDP-Man, for formation of vitamin C), GDP-L-Fucose (GDP-L-Fuc, used for cell wall formation), and ADP-based NDP-sugars such as ADP-Glucose (ADP-Glc, used to form starch) (Bar-Peled and O’Neill, 2011; Kleczkowski and Decker, 2015). However, one group stands out—the UDP-sugars. They are vital to a plant not only because of the importance of the processes they are involved in (e.g., sucrose and cell wall formation, etc.) (Figure 1) (Bar-Peled and O’Neill, 2011; Kleczkowski and Decker, 2015) but also because of their abundance (UDP-sugars may comprise up to 55% of the total nucleotide pools) (Wagner and Backer, 1992) and the profuse number of reactions where they serve as substrates. For instance, UDP-Glc alone is suggested to be involved in 270 reactions^2^ (Chae et al., 2014).

As UDP-sugars are involved in many processes which are crucial to understand plant development, but also for commercially important processes, it is of the utmost importance to understand how, where, and when these UDP-sugars are produced. This review will attempt to bring clarity to some aspects of this central part of plant metabolism.

There are several aspects to consider when discussing UDP-sugars. They include not only 1) UDP-sugar synthesis either from sugar-1-phosphates *via* distinct pyrophosphorylase reactions (Kleczkowski and Decker, 2015), or 2) from sucrose by sucrose synthase (SuSy) (Schmölzer et al., 2016), or 3) *via* interconversion from one UDP-sugar into another (Bar-Peled and O’Neill, 2011), but also 4) intracellular transport of UDP-sugars *via* specific membrane-bound transporters (Orellana et al., 2016) and, finally, 5) utilization of UDP-sugars as substrates by hundreds of specific GTs (Osmani et al., 2009) and 6) degradation of UDP-sugars by hydrolases (Muñoz et al., 2006). This paper, however, focuses only on the first aspect, namely the enzymes involved in the *de novo* synthesis of UDP-sugars from sugar-1-phosphates.

The primary synthesis of UDP-sugars is catalyzed by specific pyrophosphorylases, which use UTP and a sugar-1-P (which may be *N*-acetylated) as substrates to form inorganic pyrophosphate (PP_i_) and the corresponding UDP-sugar. This reaction is reversible (Kleczkowski et al., 2010, 2011b) and magnesium dependent (Litterer et al., 2006a; Mu et al., 2009; Yang et al., 2010), and the addition of substrates and release of products can be classified as an ordered bi-bi mechanism (Kleczkowski et al., 2011b). In plants and eukaryote parasites, the first substrate to bind is UTP, followed by sugar-1-P, which is followed by the release of PP_i_ and, subsequently, UDP-sugar (Elling, 1996; Dickmanns et al., 2011; Kleczkowski and Decker, 2015), whereas for one *Trypanosoma* pyrophosphorylase the binding order is reversed (Urbaniak et al., 2013). Plants contain three different classes of UDP-sugar metabolizing pyrophosphorylases: UDP-Glc pyrophosphorylase (UGPase), UDP-sugar pyrophosphorylase (USPase), and UDP-*N*-acetylglucosamine (UDP-GlcNAc) pyrophosphorylase **(**UAGPase). These enzymes have very low identity at amino acid (aa) level (below 20%), but they share some common structural features such as a catalytic central domain containing a Rossmann-like fold (common in nucleotide-binding enzymes), which is flanked by N- and C-terminal domains that may have regulatory roles (Kleczkowski et al., 2011b).

The work on UDP-sugar producing pyrophosphorylases has been extensively discussed by Bar-Peled and O’Neill (2011) and Kleczkowski and Decker (2015). In the present review, we will focus more on current developments and on our own research on those enzymes during the last decade or so.

## UGPase Enzyme

Following the identification of UDP-Glc by Leloir and coworkers in the 1940s (while investigating the metabolism of galactose) (Frey, 1996), a few years later yeast UGPase was discovered and described (Kalckar, 1957). Since then, many studies have shown that UGPase can utilize Glc-1-P and UTP to form UDP-Glc and PP_i_ or vice versa. Enzymes that are able to catalyze this reaction were found in both prokaryotes and eukaryotes (in the cytosol) and are denoted as UGPase-A (Kleczkowski et al., 2010; Führing et al., 2013b). In this paper, unless otherwise indicated, all mentions of “UGPase” will refer to UGPase-A. Plants also contain chloroplastic UGPase, which is commonly denoted UGPase-B (or UGPase3) (Okazaki et al., 2009; Kleczkowski and Decker, 2015).

As all UGPases carry out a freely reversible reaction, the following chapter will be started with a short summary of the known roles of the compounds that can be produced/utilized by UGPase.

### Products of Forward Reaction of UGPase: Roles of UDP-Glc and PP_i_

UDP-Glc serves as substrate for the formation of disaccharides, such as the signaling and energy transport/storage molecule sucrose or the signaling molecule trehalose-6-P (Kunz et al., 2014; Lunn et al., 2014; Li and Sheen, 2016; Andersson et al., 2018; Ciereszko, 2018). UDP-Glc itself may serve as signaling molecule (Janse van Rensburg and Van den Ende, 2018) (see below). UDP-Glc is also a precursor for the formation of important cell wall polymers, such as cellulose, callose, and hemicellulose but is also needed for the formation of different glycolipids and for glycosylation of proteins (Kleczkowski and Decker, 2015). Other role for UDP-Glc is to serve as a substrate for glycosylation of a plethora of secondary metabolites such as steroids, flavonoids, phenylpropanoids, betalains, terpenoids, and glucosinolates (Vogt and Jones, 2000; Kleczkowski et al., 2010). Glycosyl-hormone conjugates are also formed from UDP-Glc and hormones, such as auxin, cytokinin (Bajguz and Piotrowska, 2009), gibberellic acid (GA) (Piotrowska and Bajguz, 2011), salicylic acid (SA) (Rivas-San Vicente and Plasencia, 2011), and abscisic acid (Priest et al., 2005), possibly to store and stabilize the hormones (Kleczkowski and Schell, 1995).

As for PP_i_, the other product of the forward reaction of UGPase and of other NDP-sugar pyrophosphorylases, it has been suggested to be involved in controlling the directionality of the reaction, that is, drive the reversible reaction towards NDP-sugar synthesis or degradation (Igamberdiev and Kleczkowski, 2011; Sawake et al., 2015). To steer the reaction towards NDP-sugar synthesis, the PP_i_ can be used as substrate by various pyrophosphatase (PPase) enzymes to form inorganic phosphate (P_i_). Plants with reduced levels of chloroplastic PPase are containing higher levels of PP_i_ compared to wild-type (wt) plants and are less capable of starch formation (George et al., 2010). Modifications of PPase levels have also been shown to affect fruit and seed development and metabolism. For instance, overexpression of a tomato fruit cytosolic PPase led to increased ascorbate content (vitamin C) and soluble carbohydrate content together with reduced starch levels (Arias et al., 2013), whereas in *Arabidopsis thaliana* increased levels of cytosolic PPase led to low-oil phenotype in seeds and reduced levels of PPase caused an increase in seed-oil content at the expense of seed storage proteins (Meyer et al., 2012). PP_i_ has also been suggested as an alternative energy source in conditions when ATP is limited (such as anoxia) (Igamberdiev and Kleczkowski, 2011). P_i_ and PP_i_ may also be used by proton pumping PPases to maintain acidity in the vacuoles and participate in loading of sucrose to the phloem (Pizzio et al., 2015; Khadilkar et al., 2016). At least some of these effects may be in part attributed to changes in [PPi], which affect NDP-sugar production by NDP-sugar metabolizing pyrophosphorylases.

### Products of Reverse Reaction of UGPase: Roles of Glc-1-P and UTP

In plants, monosaccharides can be phosphorylated at position 1 or 6 by a hexokinase (HXK). For Glc, the HXK reaction commonly results in phosphorylation at position 6, forming Glc-6-P. This compound, being charged, has a reduced ability to traverse membranes and is thus trapped in the compartment where it was formed. Glc-6-P is an important intermediate of glycolysis (Conway and Voglmeir, 2016), but it can also be easily converted to Glc-1-P by phosphoglucomutase (PGM). Glc-1-P may also be produced by α-glucan phosphorylases from linear glucans (Stitt et al., 2010) or originate from fructose-6-phosphate (Fru-6-P), which is sequentially used by phosphoglucoisomerase (PGI) and PGM (Stitt et al., 2010) or by the breakdown of UDP-Glc *via* the reverse reaction of UGPases (Kleczkowski et al., 2010). In some heterotrophic tissues, such as barley endosperm and oilseed rape embryos, UGPase-derived Glc-1-P has been implicated in starch formation. In the proposed scheme, UGPase uses sucrose-derived UDP-Glc (produced by sucrose synthase) as a substrate to produce Glc-1-P, which will subsequently serve as substrate for ADP-Glc pyrophosphorylase (AGPase), a key regulated enzyme producing ADP-Glc for starch synthesis (Kleczkowski, 1994a, 1996; Bahaji et al., 2014; Schwender et al., 2015). In this way, there is a direct connection between sucrose breakdown and starch synthesis. Consistent with this assumption, an UGPase1 rice mutant (*flo8*), with 70% reduced UGPase activity, showed a decrease in starch content and displayed changes in starch properties (pasting, swelling) and in amylopectin structure (Long et al., 2017).

Plant synthesis of uridine originates from orotic acid (OA) or 5-phosphoribosyl-1-pyrophosphate, which in turn originates from Glc-6-P. The formed uridine monophosphate can subsequently be phosphorylated/dephosphorylated by kinases and phosphatases, which allows the UTP/UDP and ATP/ADP ratios to be equilibrated (Zrenner et al., 2006; Igamberdiev and Kleczkowski, 2011). UTP may, as mentioned before, be used for UDP-sugar synthesis, converted into cytosine triphosphate (CTP), used in RNA synthesis or, if dephosphorylated, degraded into β-alanine. Interestingly, when potato tubers were separated from the mother plant, the uridine pool rapidly decreased, whereas subsequent provision of the uridine precursor OA led to a restoration of the uridine pools, followed by an increase in sucrose mobilization, cell wall synthesis, and starch mobilization. The addition of OA to Arabidopsis plants also led to increased sucrose mobilization; however, it is unknown how/if changes in the uridine-pools influence UDP-sugar synthesizing enzymes (Loef et al., 1999; Zrenner et al., 2006).

### UGPase Gene Expression and Post-translational Modifications

In plants, there are usually at least two distinct genes coding for UGPase-A and a single gene for UGPase-B (Meng et al., 2008; Okazaki et al., 2009; Kleczkowski and Decker, 2015). Genes for UGPases-A and -B are expressed throughout the whole plant, in both source and sink tissues (Meng et al., 2009b; Okazaki et al., 2009), with the corresponding enzymes found even in vascular tissues such as phloem and xylem (Dafoe and Constabel, 2009; Beneteau et al., 2010). UGPase-B is expressed in Arabidopsis leaf, stems, and flowers and is co-expressed with sulfoquinovosyl diacylglycerol synthesis genes that are involved in sulfolipid biosynthesis, where UDP-Glc is a key precursor (Okazaki et al., 2009; Shimojima, 2011). Both types of UGPases are upregulated in response to limitations in P_i_ availability (Kleczkowski et al., 2010). UGPase-A is also transcriptionally controlled in response to several stimuli such as cold, sugars, light conditions, etc. (Gupta and Sowokinos, 2003; Kleczkowski et al., 2004; Ciereszko et al., 2005; Kleczkowski and Decker, 2015). Other carbohydrate-related processes, such as successful plant-grafts and ripening of fruits, have also been reported to be connected with changes in UGPase expression (Hu et al., 2016; Baron et al., 2016). In Arabidopsis, when considering different tissues, UGPase activity and its protein levels appeared to be related to each other, whereas the transcript levels were not closely linked, which may suggest that some type of post-transcriptional or translational regulation is involved, especially in the roots (Meng et al., 2009b). During seed filling, however, transcript levels of Arabidopsis gene for UGPase-A2 appeared to correlate with protein content, suggesting little or no post-transcriptional and translational regulation of the UGPase during this process (Hajduch et al., 2010).

Plant UGPases have been shown to be post-translationally modified in a number of ways. Examples include phosphorylation of Ser419 of sugarcane UGPase (Soares et al., 2014), binding to 14-3-3 proteins for the enzymes from barley and Arabidopsis (Alexander and Morris, 2006; Swatek et al., 2011), rice UGPase acetylation (Chen et al., 2007b), *N*-glycosylation of rice and maize UGPases (Komatsu et al., 2009; Silva-Sanchez et al., 2014), and *S*-glutathionylation, as shown in Arabidopsis cell culture during oxidative stress (Dixon et al., 2005). UGPases have also been demonstrated as sensitive to redox regulation and can interact with thioredoxins *in vivo*, as in wheat and *Medicago truncatula* seeds (Wong et al., 2004; Alkhalfioui et al., 2007). Oxidation of sugarcane UGPase by H_2_O_2_ was specifically shown to reduce its activity, whereas subsequent reduction restored the activity (Soares et al., 2014). This redox regulation was explained by possible *S*-glutathionylation caused by H_2_O_2_ oxidating thiol groups. Redox regulation was also demonstrated for UGPases from protozoan parasites *Entamoeba* and *Giardia* (Martínez et al., 2011; Ebrecht et al., 2015). However, in the case of the parasites, the redox sensitivity of UGPase involved formation of intramolecular disulfide bridge(s) between cysteines. Such a pairing of cysteines is rather unlikely for plant UGPases, due to a large distance between cysteines in crystallized Arabidopsis UGPase (McCoy et al., 2007). Recently, Euglena UGPase was also found to be redox regulated, with oxidation by hydrogen peroxide inactivating the enzyme, which could be reversed by reduction of dithiothreitol or thioredoxin (Muchut et al., 2018). The inactivation was explained by the formation of sulfenic acid derivatives by oxidized cysteines, possibly leading to a change of conformation of the protein.

### UGPase Structure

Molecular masses of plant UGPase-A proteins are usually in the range of 50–55 kDa, depending on plant species (Sowokinos et al., 1993; Meng et al., 2008), and they are much smaller than the ca. 90 kDa mature UGPase-B protein (Okazaki et al., 2009). Whereas not much is known about the structure of UGPase-B, there is relative abundance of information on UGPase-A, which has been intensively studied by site-directed mutagenesis (both in plants and animals) and has been crystallized from several species (Kleczkowski et al., 2010). Arabidopsis and sugarcane UGPases-A, the only plant enzymes of this type that had their structures solved (McCoy et al., 2007; Cotrim et al., 2018), contain three major domains: 1) a large central domain, which includes a sugar-binding loop, flanked by 2) a N-terminal domain, which contains a nucleotide-binding loop, and 3) a C-terminal domain, which mainly consists of a β-helix type of fold; such β-helices are reported to increase enzyme stability, for example, in pectate/pectin lyases and polygalacturonases (D’Ovidio et al., 2004). This structural blueprint is generally similar for UGPases from different eukaryotic sources (Roeben et al., 2006; Steiner et al., 2007; Kleczkowski et al., 2011b).

In addition to crystal structure analyses, the interactions between UGPase and its substrates were also investigated by site- and motif-directed mutations/deletions (Katsube et al., 1991; Martz et al., 2002; Geisler et al., 2004; Meng et al., 2009a). Deletions in the UGPase N-terminal domain (37 aa) led to decreased affinity for UDP-Glc (increased Michaelis constant, K_m_), whereas deletions in the C-terminal domain (from 8 aa, up to 101 aa) led to decreased affinity for PP_i_ (Meng et al., 2009a). UDP-Glc binding to UGPase led to a displacement of the C-terminal β-helix-containing domain towards the substrates which may be critical for PP_i_ binding (McCoy et al., 2007; Meng et al., 2009a).

**Figure 2:**
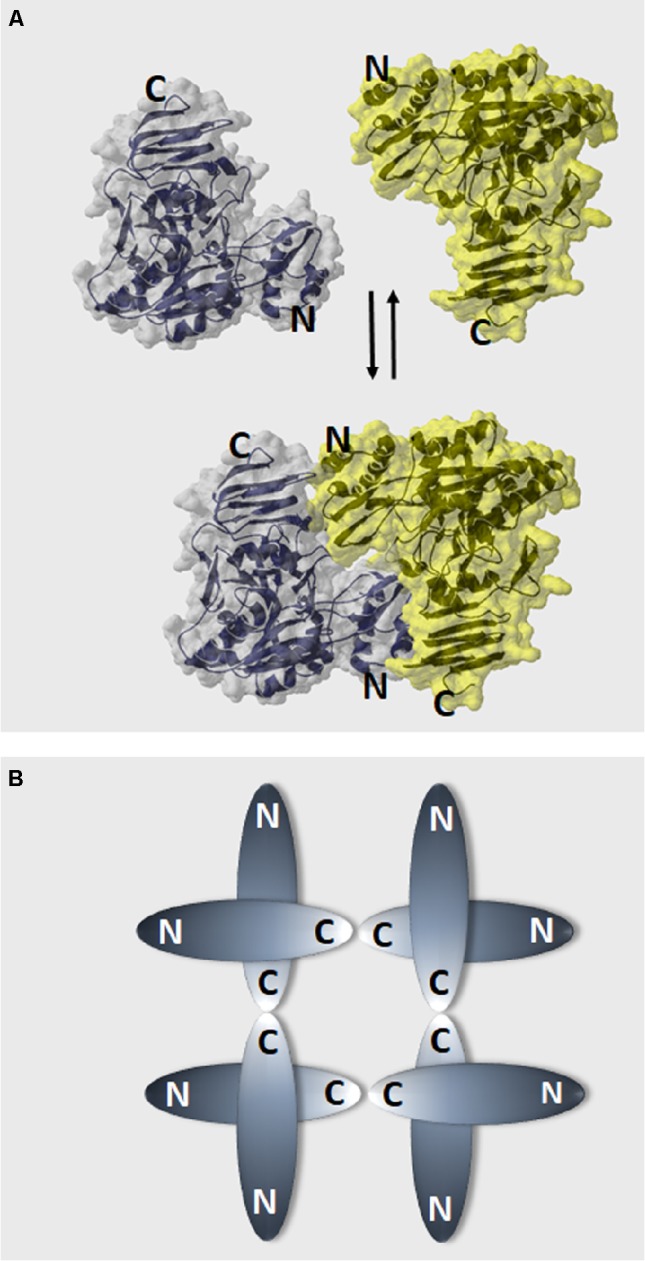
Models of plant UGPase dimer formation and the oligomerization state of human and yeast UGPases. **(A)** “Head-to-toe” dimer of Arabidopsis UGPase (McCoy et al., 2007), where N-terminal domain of one monomer faces C-terminal domain of the other. Surface and ribbon representations (PDB code: 2ICX) were built using Swiss PDB viever. **(B)** A schematic drawing of the “toe-to-toe” arrangement of the human and yeast UGPase octamer, where oligomerization occurs *via* interaction of the C-termini domains of the monomers (Roeben et al., 2006; Führing et al., 2013b).

An important aspect in which plant (and protozoan parasites) UGPases distinguish themselves from the UGPases of other eukaryotes (human and yeast) is in their oligomerization mode. Human and yeast UGPases are active as octamers (Roeben et al., 2006; Führing et al., 2013a,b), whereas the monomer is the only active form of plant and parasitic UGPases (Martz et al., 2002; McCoy et al., 2007; Steiner et al., 2007; Kleczkowski et al., 2011b; Martínez et al., 2011; Ebrecht et al., 2015). When the crystal structure of Arabidopsis UGPase-A1 was resolved, the protein was observed as a mixture of monomers and dimers, with the latter formed by the “head-to-toe” positioning of adjoined monomers, that is, N-terminal of one monomer facing C-terminal domain of the other. In this way, active sites on the monomers may be hindered, thus inactivating the UGPase (Figure 2A) (McCoy et al., 2007).

The monomer-to-dimer conversion is thought to be regulated by subtle changes in hydrophobicity in the immediate vicinity of the protein, by the concentration of UGPase protein, and by substrates/products of its reaction (Kleczkowski et al., 2005; Decker et al., 2012). The effect of substrates, determined using Gas-phase Electrophoretic Mobility Macromolecule Analysis (GEMMA) (Decker et al., 2012), revealed that addition of substrates/products of UGPase reaction resulted in a shift from higher order oligomers to the monomers. As active site binding of Glc-1-P and PPi needs prior binding of UTP and UDP-Glc, respectively, and the active site of the UGPase in the dimer would be hindered (assuming the “head-to-toe” configuration of the monomers), binding of the substrates to the active site would require their prior binding to an independent allosteric site (or sites) for the substrates (Decker et al., 2012; Führing et al., 2013b). Such a site is to date unknown, but this may represent a plant-specific mechanism (Führing et al., 2013b). Magnesium was not included during GEMMA studies; however, for human UGPase, it has been suggested to be required only for catalysis of the reaction but not essential for substrate binding (Führing et al., 2013a).

An alternative model for oligomerization was later proposed for sugarcane UGPase, based on small-angle X-ray scattering analyses of the purified protein in solution (Soares et al., 2014). The results suggested that sugarcane UGPase forms dimers and higher order oligomers exclusively *via* interaction of C-termini of the monomers (toe-to-toe configuration), similarly to the arrangement of subunits in human and yeast UGPase ( Figure 2B). The formation of such a dimer could induce conformational changes, which may affect activity (Soares et al., 2014). This type of dimer would also allow substrates to directly interact with the active site, which may subsequently affect the oligomerization status. It would also more closely resemble the dimer structure observed in other eukaryote UGPases such as human or yeast, although these require the formation of higher order oligomers for activity (Roeben et al., 2006; Führing et al., 2015). Interestingly, earlier studies on barley UGPase (Meng et al., 2009a) have revealed that the very end of its C-terminal domain (final 8 aa) was essential for dimer formation. Deletion of those 8 aa led to a solely monomeric protein, which was 40% more active than the wt enzyme, supposedly by increasing the amounts of active monomers (Meng et al., 2009a). Removal of longer regions of the C-terminal domain resulted in inactivation of plant UGPases (Woo et al., 2008; Meng et al., 2009a).

More recent studies on sugarcane UGPase, based on crystal structure analyses of the purified enzyme (Cotrim et al., 2018), confirmed that the protein indeed exists as a mixture of monomers and higher order oligomers. However, the structural arrangement of subunits in a dimer resembled the “head-to-toe” structure described for Arabidopsis UGPase (McCoy et al., 2007), although details of the subunit interaction were different. It is unclear why analyses of sugarcane UGPase dimers in solution differ from those obtained for crystallized protein, and the exact mechanism of the (de)oligomerization of plant UGPases (and its possible *in vivo* roles) requires further studies.

### UGPase Subcellular Localization

Plant UGPase protein (and activity) has been shown to be localized mostly in the cytosol but is also present in other cell compartments, including chloroplasts (Kleczkowski et al., 2010; Wan et al., 2016). In the chloroplast, UGPase-B type is responsible for a large portion of the observed UGPase activity (Okazaki et al., 2009). For UGPase-A, immunolabeling of cell fractions from rice cell culture has shown that it was predominantly localized not only in the cytosol but also to some extent in the amyloplasts and Golgi (Kimura et al., 1992) as well as in microsomal fractions (Mikami et al., 2001). Microsomal fractions of the dicotyledonous plants such as tobacco, potato, and Arabidopsis were also shown to contain UGPase-A protein (Mikami et al., 2001; Boher et al., 2013; McLoughlin et al., 2013). UGPase activity has been observed in membrane fractions from young leaves and coleoptiles in barley (Becker et al., 1995). However, whether this activity belongs to UGPase, USPase, and/or UAGPase is unknown at present (all three enzymes may use UDP-Glc as substrate).

Despite the above-mentioned reports of membrane-associated UGPase (or UGPase-like) activity, the enzyme is most likely not an integral part of cellular membranes. Based on the details of UGPase protein structure (McCoy et al., 2007) as well as on the results of two web-based prediction programs (using the UGPase aa sequence as query), the protein lacks any transmembrane domains (von Heijne, 1992; Hofmann and Stoffel, 1993). Thus, it seems likely that the UGPase membrane association occurs *via* interaction with some membrane proteins or with the cytoskeleton. For instance, membrane-bound UGPase was found to associate with *PLASMODESMATA-LOCATED PROTEIN 1*, which directs callose deposition during downy mildew infection of Arabidopsis (Caillaud et al., 2014). Whether or not the different plant UGPase isozymes are differently localized and/or have different affinity for membrane association is unknown. It should be also pointed out here that post-translational modification has been reported to affect association with specific membranes for yeast UGPase and for plant SuSy (Amor et al., 1995; Cardon et al., 2012).

### Studies on *in vivo* Roles of UGPases

**Figure 3:**
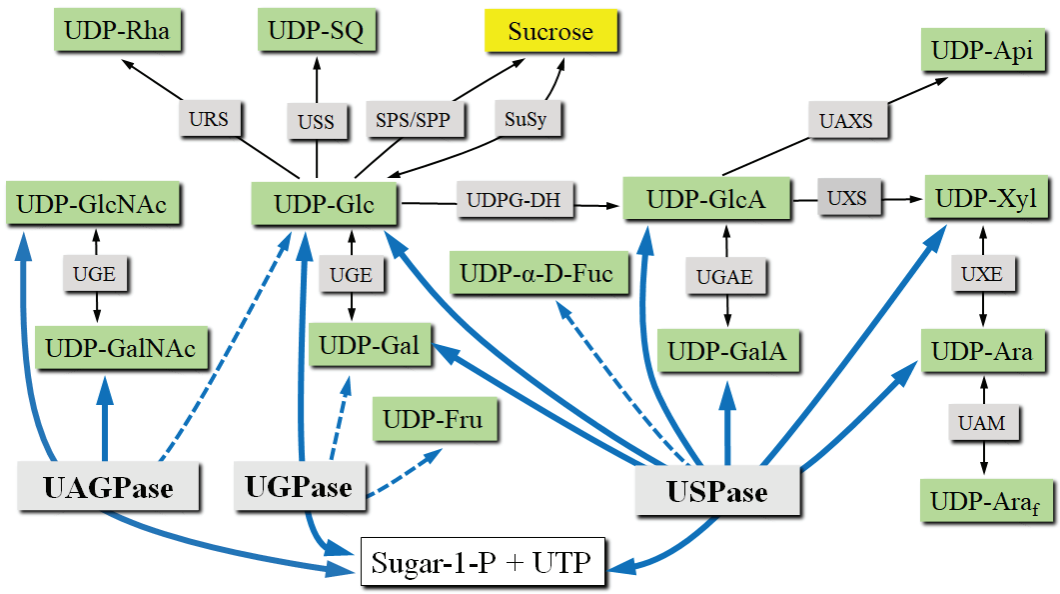
UDP-sugar formation and interconversion network. Green boxes show selected UDP-sugars; gray boxes show enzymes involved in UDP-sugar formation or interconversion. Intracellular compartments and UDP-sugar transporters are omitted for simplicity. Blue lines connect respective substrates and products of UGPase, USPase, and UAGPase. Dotted lines refer to less specific reactions. *Abbreviations*: SPP, sucrose phosphate phosphatase; SPS, sucrose phosphate synthase; SuSy, sucrose synthase; UAGPase, UDP-GlcNAc pyrophosphorylase; UAM, UDP-Ara mutase; UAXS, UPD-apiose/UDP-Xyl synthase; UDPG-DH, UDP-Glc dehydrogenase; UGAE, UDP-GlcA epimerase; UGE, UDP-Glc epimerase; UGPase, UDP-Glc pyrophosphorylase; URS, UDP-rhamnose synthase; USPase, UDP-sugar pyrophosphorylase; USS, UDP-sulfoquinovose synthase; UXE, UDP-Xyl epimerase; UXS, UDP-Xyl synthase.

Several studies were carried out on transgenic plants, both mono- and dicotyledonous, where expression of UGPase(s) was reduced or knocked out. Plants lacking the expression of chloroplastic UGPase-B in Arbidopsis retained approx. 40% chloroplastic UGPase activity of unknown source but were completely devoid of sulfolipids in their chloroplast membranes (Okazaki et al., 2009), underlining the crucial role of this enzyme in the formation of sulfoquinovose, a Glc-derived constituent of sulfolipids. On the other hand, studies on plants with reduced or knocked out expression of cytosolic UGPase-A were less successful in revealing the exact roles of this protein. RNA interference against rice (*Oryza sativa*) UGPase1 and 2 led to significantly smaller plants (40 and 30% reduction in stature, respectively) (Chen et al., 2007a; Woo et al., 2008), whereas a double knockout of UGPase1 and UGPase2 (achieved using T-DNA inserts) in Arabidopsis resulted in dwarf plants, possibly caused by a reduction in cell size (Park et al., 2010). These plants retained ca. 6% of UGPase-like activity (Glc-1-P dependent), probably representing activities of USPase, UAGPase, and chloroplastic UGPase-B see (Figure 3), Similarly, when UGPases (of the UGPase-A type) were removed from leaf crude extracts by immunoprecipitation with UGPase antibodies, ca. 10% of UGPase-like activity remained (Kleczkowski and Decker, 2015). Stem lengths in Arabidopsis plants with UGPase1 and UGPase2 activities knocked down to ca. 25% were unaffected (Meng et al., 2009b), which may suggest that a threshold of UGPase activity for normal growth exists within the 6–25% range of wt UGPase activity (Park et al., 2010). This may, however, differ in various tissues and species, as for example in transgenic potato tubers, where a 96% reduction in UGPase activity had no effect on the development of the tubers (Zrenner et al., 1993). Interestingly, *At*UGPase1 was crucial for activating, *via* an unknown but possibly structural mechanism, a fumosin (a sucrose-related bacterial signal) induced programmed cell death (PCD), by affecting the machinery for photosynthesis, cellular detoxification and *via* effects on the levels of β-carbonic anhydrase (Chivasa et al., 2013).

The most striking effects of the reduction of UGPase activity concerned the development of the male gametophytes (pollen). Reduced expression of rice UGPase1 (caused by co-suppression from an overexpression construct) led to aborted pollen, due to altered callose deposition (Chen et al., 2007a). Similarly, an intron-bordering point-mutation in rice UGPase1, which resulted in truncated and inactive protein, also led to plants that were male-sterile (Woo et al., 2008). Reductions in expression of rice UGPase2 led to decreased starch deposition in the final stages of pollen development, which again resulted in reduced pollen viability (Mu et al., 2009). In contrast to rice UGPases, studies on transgenic Arabidopsis plants where UGPase genes were individually inactivated revealed no effects on pollen development (Meng et al., 2009b; Park et al., 2010). However, knockouts of UGPase1 in Arabidopsis exhibited shorter and aborted siliques and an increased seed abortion (Fusari et al., 2017). Knocking out both UGPase1 and UGPase2 interfered with callose deposition and led to pollen abortion. Interestingly, this male sterility could be prevented by supplementation of 1.5% UDP-Glc to the plant growth media (Park et al., 2010). It is unknown whether these differences in the importance of UGPase isoforms between Arabidopsis and rice reflect differences in UGPase isoform stoichiometry or are due to differences between monocot and dicot pollen development or to some other mechanism.

Several studies have also attempted to determine the roles of UGPase *in vivo* by using an overexpression strategy. Thus, overexpression of plant or bacterial UGPase in different poplar species resulted in plants with reduced stature and leaf area or weight; the studies, however, presented conflicting data with regard to the effects on lignin content (reduced vs. unaltered and increased vs. unaltered S/G ratios) and phenolic glucosides (a 233-fold increase in salicylic acid (SA)-2-*O*-glucoside alone vs. a 2.7-fold increase coupled with a 1.4- to 2.6-fold increase in other phenolic glucosides) (Coleman et al., 2007; Payyavula et al., 2014). Overexpression of UGPase in dicotyledonous species, such as Arabidopsis, jute, and cotton (overexpression using highly active 35S promoters fused to cDNA of cotton or *Larix* UGPase in Arabidopsis and of the native UGPases for jute and cotton), led to plants with a higher cellulose content and longer stems (all studies) and higher sucrose content (2 of the studies) (Wang et al., 2011; Zhang et al., 2013; Li et al., 2014, 2015). On the other hand, under the control of a “weaker” ubiquitin promoter, sorghum UGPase overexpression in Arabidopsis failed to cause any obvious phenotypes (Jiang et al., 2015). With those and similar overexpression studies, it should be emphasized that, while overexpression of a given protein may highlight its capability to be involved in a given pathway, this does not necessarily constitute a proof of its *in vivo* function. In this respect, studies on plants with reduced or knocked out activity of the studied enzyme are more revealing.

In summary, as evident from examples given above, whereas the *in vivo* role of chloroplastic UGPase-B, as an important player in sulfolipid biosynthesis (Okazaki et al., 2009), appears to be established, the roles of cytosolic UGPases (of the UGPase-A type) are less clear. The latter has certainly a role during plant male reproductive phase, but their exact functions *in vivo* remain somewhat elusive.

## USPase Enzyme

In comparison to UGPase, USPase was only relatively recently identified in pea and Arabidopsis plants (Kotake et al., 2004; Litterer et al., 2006b; Kotake et al., 2007). There are, however, several older studies which described assays of crude plant extracts for enzyme activities resembling those of USPase, but attributed to other enzymes (Kleczkowski et al., 2011a). USPase enzymes have been identified in most plant species (excluding some red algae), eukaryote parasites, and a few bacterial species, but no vertebrate USPases have been reported (Gross and Schnarrenberger, 1995; Kleczkowski and Decker, 2015). USPase commonly catalyzes the reversible conversion of a wide range of sugar-1-phosphates (mainly Glc-1-P, Gal-1-P, GlcA-1-P, GalA-1-P, L-Ara-1-P, Xyl-1-P) and UTP to the corresponding UDP-sugar and PP_i_ see (Figure 3). Because of this substrate promiscuity, the enzyme is sometimes denoted as “Sloppy” (Bar-Peled and O’Neill, 2011). A systematic screen of Arabidopsis USPase activity against a range of possible sugar-1-phosphates as substrates revealed that, besides the sugar-1-phosphates mentioned above, the Arabidopsis enzyme reacted also with α-D-Fuc-1-P (Km of 3.4 mM) (Decker and Kleczkowski, 2017). This is in contrast to barley USPase which had no activity with α-D-Fuc-1-P (Wahl et al., 2017), indicating differences in substrate specificities between Arabidopsis and barley USPases.

### Roles of UDP-Gal and Gal-1-P

UDP-galactose (UDP-Gal) is used for the synthesis of galactolipids, such as monogalactosyldiacylglycerol and digalactosyldiacylglycerol, which constitute a large portion of the chloroplast membranes (Dörmann and Benning, 2002; Kelly and Dörmann, 2002). Galactose originating from UDP-Gal is deposited in the primary cell walls as component of hemicelluloses, such as galactomannans (Pauly et al., 2013), and of pectins, such as rhamnogalacturonan I (RGI) and rhamnogalacturonan II (RGII) sidechains (Bacic, 2006; Loque et al., 2015). UDP-Gal is also used as precursor for galactinol, which together with sucrose is used for the synthesis of polysaccharides (raffinose and stachyose) (Kleczkowski et al., 2011a) and to form the backbone of arabinogalactans for protein modification (Nothnagel, 1997). In plants, UDP-Gal could also be used to form hormone conjugates such as auxin-galactoside (Corcuera et al., 1982) and secondary metabolites such as triterpenoid saponin-galactoside (Shibuya et al., 2010).

Free galactose can be converted to Gal-1-P by anomeric galactokinases (Egert et al., 2012); both compounds are however generally toxic to cellular metabolism, and they are usually converted to less harmful compounds *via* the classical Leloir-pathway (Lang and Botstein, 2011). During most developmental stages, higher plants appear to lack or have a limited activity of Gal-1-P uridylyltransferase (GALT), the key enzyme of the Leloir-pathway (Frey, 1996; Dai et al., 2006), for example, the closest Arabidopsis GALT homolog (At5g18200) has been shown to function rather as a ADP-Glc phosphorylase (McCoy et al., 2006). Thus, in plants, USPase may well serve as an alternative Gal-1-P removal mechanism (Isselbacher pathway), producing UDP-Gal for glycosylation reactions.

### Roles of UDP-GalA, UDP-GlcA, GalA-1-P, and GlcA-1-P

UDP-galacturonic acid (UDP-GalA) is used as substrate for synthesis of the backbones of both unbranched (homogalacturonan) and branched types of pectin (RGI and RGII) (Usadel et al., 2004; Bacic, 2006), and it contributes to the reducing ends on the glucuronoxylan (hemicellulose) (Rennie and Scheller, 2014). UDP-glucuronic acid (UDP-GlcA) provides GlcA-units to the glucuronoxylan (Pauly et al., 2013; Rennie and Scheller, 2014) and to pectin RGII (Bacic, 2006). UDP-GlcA is also used in the formation of flower pigments (Sasaki et al., 2005; Sawada et al., 2005). Both GlcA-1-P and GalA-1-P can be produced by anomeric kinases (Yang et al., 2009; Geserick and Tenhaken, 2013a), while GlcA-1-P can also originate from the *myo*-inositol oxygenation pathway (Geserick and Tenhaken, 2013b).

### Roles of UDP-Xyl, UDP-Ara, UDP-α-D-Fuc, Xyl-1-P, and Ara-1-P

UDP-xylose (UDP-Xyl) provides xylose for the backbone of the xylans (Rennie and Scheller, 2014), and UDP-Xyl-derived xylose may also be present in different domains of pectin such as RGII and xylogalacuronan (Bacic, 2006; Scheller et al., 2007). A specific GT (which uses UDP-Xyl but not UDP-Glc) was shown to produce xylosyl-cytokinin conjugates (Martin et al., 1999). Glycoproteins may also be formed with UDP-Xyl as substrate (Kleczkowski et al., 2011a). Arabinose originating from UDP-arabinose (UDP-Ara) can be found in the branched pectins (Bacic, 2006) and in hemicellulose in grasses and some dicotyledonous species (not Arabidopsis and poplar) (Rennie and Scheller, 2014). No xylose-kinase has yet been identified, but anomeric kinases which phosphorylate L-Ara have been reported (Yang et al., 2009; Geserick and Tenhaken, 2013b). Fuc moiety, usually in the form of α-L-Fuc rather than β-L-Fuc, is found in cell wall polysaccharides and in sugar modules of glycoproteins (Scheible and Pauly, 2004), where it is inserted by inverting type of a glycosyltrasferase, which uses GDP-β-L-Fuc as substrate and inverts anomeric configuration of the sugar residue upon the transfer (Lairson et al., 2008). There is no apparent use for UDP-α-D-Fuc, unless some unknown type of a glycosyltransferase can use it as substrate (Decker and Kleczkowski, 2017).

### USPase Structure

The USPase gene encodes a protein of ca. 67–70 kDa (Kotake et al., 2004; Litterer et al., 2006a,b). Since no plant USPase has been crystallized, their structures could only be inferred from homology models done on *Leishmania major* (a eukaryotic single-celled pathogen) USPase, which had its structure solved (Dickmanns et al., 2011). The protein is active as a monomer and, unlike plant UGPase, there is no evidence for oligomerization (Damerow et al., 2010). The *Leishmania* USPase has generally a similar structure to UGPase-A or UAGPase proteins and is composed of central domain with prominent Rossmann fold and containing the active site flanked by characteristic N- and C-terminal domains (Dickmanns et al., 2011). The active site of USPase is larger than in other UDP-sugar metabolizing pyrophosphorylases, probably reflecting the relative non-specificity of this enzyme with respect to sugar-1-P as substrate (Dickmanns et al., 2011; Kleczkowski et al., 2011b).

### USPase Expression, Subcellular Localization, and Tissue Specificity

Arabidopsis USPase is expressed in most young plant tissues, with stronger expression observed in the roots, cotyledons, and vascular tissues, but the most prominent expression was found in the flowers and, more specifically, in the developing pollen (Litterer et al., 2006b; Kotake et al., 2007). In Arabidopsis knockdowns with reduced UGPase activity, the expression of USPase gene was upregulated in flowers, roots, and in mature (but not young) leaves, probably representing a compensatory mechanism for UGPase silencing (Meng et al., 2009b). Such a regulation of the expression of USPase appears not only connected to UGPase expression but may also be controlled together with the expression of GlcA-kinase which supplies GlcA-1-P, the substrate for USPase (Geserick and Tenhaken, 2013a).

In Arabidopsis florets, USPase protein was located mainly in the cytosol, but also some portion was found in the microsomal fraction. When all USPase enzymes were removed from protein samples originating from these florets (using antibodies), no residual UDP-GlcA pyrophosphorylation activity could be observed, suggesting that USPase is the major enzyme that uses/produces GlcA-1-P in this organ. Immunoblots of natively extracted USPase enzyme revealed two separate bands, and these were proposed to represent either two separate isoforms or differentially post-translationally modified versions of the USPase enzyme (Gronwald et al., 2008).

### Studies on *in vivo* Roles of USPase

A number of studies have attempted to use transgenic plants with altered levels of USPase to decipher the *in vivo* roles of USPase. Overexpression of Arabidopsis USPase under the control of the 35S promoter resulted in Arabidopsis plants with 143–255% increase in USPase activity (Kotake et al., 2007), but no further phenotypic differences from wt plants were observed. Similarly, Arabidopsis plants overexpressing sorghum USPase under the control of the polyubiquitin promoter were also wt-like (Jiang et al., 2015).

Whereas several studies resulted in plants with reduced levels of USPase activity, no full USPase knockout plants have been produced to date. Using Arabidopsis with T-DNA inserts in the USPase genes (lines: salk015903 and sail_223_b12), it was found (Schnurr et al., 2006) that although these plants had reduced levels of USPase transcripts and 50% USPase activity (compared to wt), no visual phenotypes or changes in the rosette cell wall content could be observed. Furthermore, selfed plants failed to produce seeds that were homozygous for the USPase T-DNA inserts, and an effect on the development of pollen was observed. The study showed that the pollens which received the mutated USPase-allele (containing the T-DNA insert) were aborted (Schnurr et al., 2006). In a subsequent study (Kotake et al., 2007) with Arabidopsis plants having USPase levels reduced to 25 and 21% (caused by antisense and co-suppression of the native USPase gene), no changes in phenotypes were observed. And again, the authors were unable to produce plants with homozygous T-DNA inserts in the USPase gene (line: salk015903) (Kotake et al., 2007).

In an attempt to circumvent the pollen abortion caused by mutated USPase alleles, the heterozygous USPase T-DNA insert plants were complemented with USPase::GFP fusions expressed under the control of different constitutive, inducible, or tissue-specific promoters (Geserick and Tenhaken, 2013b). Only plants with an USPase::GFP fusion under the control of the ubiqutin 10 promoter were able to form seeds which were homozygous for the T-DNA insert in the native USPase gene. These USPase knockdown plants retained approx. 3% USPase activity, and their anther development was affected, both by the production of shorter anthers and by problems in release of pollens from the pollen sac. These plants set seeds at a very low frequency (below 0.1% of wt), and their vegetative growth was also impaired. Most importantly, in comparison to wt, leaf extracts from USPase-knockdown plants contained higher contents of arabinose and xylose (and not glucuronic acid). This strongly suggested a role for USPase in salvaging sugars (especially arabinose and xylose) released during cell wall turnover. The sugars, thus, can be phosphorylated by a kinase (to a sugar-1-P) and then “activated” by USPase to a corresponding UDP-sugar, which can be used for synthesis of new cell wall polysaccharides (Geserick and Tenhaken, 2013b).

Overall, the results of the above-mentioned studies on transgenic plants impaired in USPase gene expression clearly pointed out to the fact that no plants with full knockout of USPase activity could be obtained. Given that USPase is able to directly form (or utilize) a plethora of different UDP-sugars, which can be used for cell wall biosynthesis or other glycosylation reactions, it is crucial to consider USPase also when studying other UDP-sugar metabolizing enzymes see (Figure 3). Thus, more efforts are required to understand in detail *in vivo* roles of USPase.

## UAGPase Enzyme

UDP-GlcNAc pyrophosphorylase (UAGPase) activity was first identified in 1954 and has subsequently been found in many species such as bacteria, human, *Drosophila*, yeast, plants, etc. (Yang et al., 2010; Chen et al., 2014). Plants usually contain two isozymes of UAGPase (each coded by distinct gene), both of them able to metabolize *N*-acetylated UDP-sugars (and PP_i_ from/to the corresponding *N*-acetylated sugar-1-P and UTP). Whereas the UAGPase1 isozyme is strictly using GlcNAc-1-P and GalNAc-1-P as the only substrates to produce the corresponding UDP-sugarNAc products, UAGPase2 can also use Glc-1-P to produce UDP-Glc (see Figure 3) (Yang et al., 2010).

### Roles of UDP-GlcNAc, UDP-GalNAc, GlcNAc-1-P, and GalNAc-1-P

The *N*-acetylated (NAc) UDP-sugars have long been studied in many eukaryotic species, not only plants. A reason for this was that UDP-GlcNAc is a well-known substrate for synthesis of chitin, an essential component of cell walls in fungi and of exoskeletons of arthropods and insects, but not in plants (Furo et al., 2015). Both UDP-GlcNAc and its C4 epimer UDP-GalNAc have been identified *in planta* (Alonso et al., 2010), and as they do not appear to contribute to bulk cell wall formation (Bar-Peled and O’Neill, 2011), they may mainly be involved in protein glycosylation (Niemann et al., 2015).

UDP-GlcNAc is a substrate for protein *N*- and *O*-glycosylation-reactions, which were shown to have roles in human diseases, for example, diabetes and prostate cancer (Ruan et al., 2013; Itkonen et al., 2014). In plants, the formation of similar protein modifications, such *N*-linked glycans and glycosylphosphatidylinositol (GPI)-anchoring, requires a network of “activated” (with UDP-, GDP- and dolichol) Glc, GlcNAc, and Man as well as the involvement of GTs (A, B and C-type) (Rayon et al., 1999; Chae et al., 2014; Kinoshita, 2014; Strasser, 2016). In wt Arabidopsis, the largest observed GlcNAc oligomers (dimers) probably originate from degradation of such *N*-linked glycans (Vanholme et al., 2014).

Plant *O*-type GlcNAc and GalNAc modifications of proteins have been shown/suggested as important for different cellular responses (Niemann et al., 2015). For example, GTs such as SPINDLY (SPY), which is *O*-linked GlcNAc transferase, can modify target proteins, playing an important role for a number of hormone-related responses. SPY maintains suppression of the GA-response, and thus, reduced levels of SPY lead to a constitutive activation of GA-responses (e.g., during embryo development) but also reduced perception of cytokinin (which may cause, e.g., senescence, increased root elongation, etc.) (Olszewski et al., 2010). It also seems interesting to note that bacterial lipid A synthesis originates from UDP-GlcNAc. Many of the lipid A precursors are synthesized in plant mitochondria, but their roles are still unknown (Li et al., 2011).

Both UDP-GlcNAc and UDP-GalNAc are transported to the endoplasmic reticulum, where protein glycosylation occurs, *via* the nucleotide-sugar transporter ROCK1. Reducing this transport leads to increased levels of cytokinin, supposedly by reducing the activity of cytokinin-degrading enzymes or targeting these for degradation; this results in increased floral meristem activity. ROCK1 was also suggested to have a role in the formation of the pollen outer wall (exine) (Niemann et al., 2015). Interestingly, a bifunctional barley UDP-Gal epimerase was characterized, which can interconvert UDP-GalNAc and UDP-GlcNAc (Zhang et al., 2006), although it is not known if this has any biological significance. GlcNAc-1-P, the substrate of UAGPase, can either be provided by the *de novo* synthesis pathway (originating from Fru-6-P) or by the salvage pathway (phosphorylation of GlcNAc by a specific kinase (*At*GNK). GalNAc may also be used by *At*GNK to form GalNAc-1-P, but at very low rates (Furo et al., 2015).

### UAGPase Structure

The two isozymes of Arabidopsis UAGPase have molecular masses of ca. 58 kDa each (Yang et al., 2010) and exist as monomers in solution. In contrast, human analogue of UAGPase (AGX1) is a dimer in conditions which resemble native environment but dissociates to active monomers under assay conditions (Peneff et al., 2001). Thus, similar to plant UGPase-A, human AGX1 and/or plant UAGPases may also be regulated by oligomerization. Protein structures of Arabidopsis UAGPase1 and UAGPase2 were homology-modeled using crystal structure of human AGX1 as template, revealing a conserved catalytic fold in the central domain and helped to identify key conserved motifs (Yang et al., 2010). Overall, the tertiary structure of UAGPase appears to resemble those of UGPase and USPase (Kleczkowski et al., 2011b).

### UAGPase Localization and Tissue Specificity

No experimental data regarding the subcellular localization of plant UAGPases exist, but predictions based on their aa sequences (SUBAcon) suggest that both UAGPase isozymes reside in the cytosol (Hooper et al., 2014). Promoter and GUS fusion studies in Arabidopsis showed UAGPase1 expression in mature pollen, stipules, and root tips, whereas UAGPase2 was expressed not only in stipules and root tips but also in immature anthers, lateral root primordia, and in floral meristems (Chen et al., 2014). Arabidopsis UAGPase2 expression could be induced by virus infections (Whitham et al., 2003). Intriguingly, using grafting of Arabidopsis plants from different ecotypes, it was shown that the UAGPase2 transcripts move *via* the phloem in a unidirectional manner from shoot to root, under full nutrient conditions (non-limiting nitrogen and phosphorus) (Thieme et al., 2015).

### Studies on *in vivo* Roles of UAGPase

There have only been a few studies that used transgenic plants to examine UAGPase roles *in vivo*. Overexpression of both of the UAGPase isozymes from *Sorghum bicolor* in Arabidopsis under control of the ubiquitin promoter led to diverse outcomes. *Sb*UAGPase1 overexpression had no visible effects, while *Sb*UAGPase2 overexpression led to not only increased biomass production and longer roots but also earlier flowering (Jiang et al., 2015).

A rice natural mutant (displaying “spotted leaf” phenotype) was recently identified as a knockout in UAGPase1 gene. Those plants exhibited early senescence, increased levels of reactive oxygen species (ROS), and constitutively activated defense response against bacterial blight (Wang et al., 2015). In Arabidopsis plants, inactivation of both of UAGPase genes was lethal, but there were also problems with producing plants with just one UAGPase gene fully inactivated (Chen et al., 2014). Plants with UAGPase1 knocked out and with only one functional UAGPase2 allele were able to form mature gametes; however, seeds with a non-functional UAGPase2 allele were subsequently aborted during embryogenesis. Plants with UAGPase2 knocked out and with a single functional UAGPase1 allele failed to develop both male and female gametes. Thus, both UAGPase isozymes were required for gametogenesis and embryo development. Interestingly, the aborted pollens formed a thicker intine wall than the wt pollens (Chen et al., 2014).

Recently, using chemical mutagenesis, a novel allelic mutant of UAGPase2, displaying “spotted” phenotype, was obtained in rice (Xiao et al., 2018). The mutant accumulated high levels of ROS, which were typical of PCD. The plants also accumulated abnormally high levels of UDP-Glc, which was attributed to the lack of UAGPase. The inability of the mutant to metabolize UDP-Glc was proposed to represent an important factor in ROS accumulation and subsequent PCD (Xiao et al., 2018). An excessive accumulation of UDP-Glc in the UAGPase2 mutant is surprising, given that the enzyme is thought to produce/utilize mostly UDP-GlcNAc and/or UDP-GalNAc rather than UDP-Glc (Kleczkowski et al., 2010; Yang et al., 2010; Kleczkowski and Decker, 2015; Decker and Kleczkowski, 2017). Also, in crude leaf extracts, it is UGPase which contributes at least 90–94% of the UDP-Glc-dependent activity, based on UGPase mutant studies (Park et al., 2010) and after immunoprecipitation with UGPase antibodies (Kleczkowski and Decker, 2015). Thus, the excess of UDP-Glc in UAGPase2 mutant may perhaps reflect an indirect effect of the mutation on UGPase *via* an unknown mechanism.

Overall, it appears thus that, similar to studies on UGPase-A and USPase in transgenic plants (at least in Arabidopsis), fertility problems of the obtained mutants may prevent more detailed analyses of non-gametophyte and non-embryogenesis-related *in vivo* role of a given UAGPase protein/gene. It was obvious, though, that the studies pointed out not only to essential roles of the UAGPases during Arabidopsis flower development/embryogenesis, senescence, and pathogen resistance but also to the role of UAGPase2 in plant PDC.

## Alternative Sources of UDP-Sugars

### Sucrose Synthase

The retaining GT-B enzyme sucrose synthase (SuSy) (Zheng et al., 2011) is generally present in plants as several isozymes, that is, six genes in *Arabidopsis thaliana* and rice (Kleczkowski and Decker, 2015) and at least seven genes in poplar (Meng et al., 2007; Gerber et al., 2014). These enzymes are able to catalyze the reversible conversion of UDP and sucrose into UDP-Glc and fructose (EC. 2.4.1.13), but it was also demonstrated *in vitro* that in this reaction either the UDP or the fructose can be replaced with other NDPs or sugars to form the corresponding NDP-sugars (forward reaction) or di-sugars (reverse reaction) (Römer et al., 2004; Sauerzapfe et al., 2008; Kleczkowski and Decker, 2015). For some of these substrates, the catalytic rate of potato SuSy was varying, strongly depending on the expression system for the recombinant SuSy (eukaryotic yeast compared to prokaryotic *E. coli*), possibly suggesting that a post-translational modification might be involved in controlling the substrate specificity of SuSy (Sauerzapfe et al., 2008). In cotton and maize, it was shown that phosphorylation of SuSy may alter its subcellular localization from plasma membrane-associated (for non-phosphorylated SuSy) to the cytosol (phosphorylated SuSy) (Amor et al., 1995; Winter and Huber, 2000).

Over twenty years ago, it was suggested that SuSy was an important contributor of UDP-Glc to the synthesis of cell wall components such as cellulose (Amor et al., 1995; Haigler et al., 2001). For instance, in bean epicotyls, SuSy was demonstrated to bind directly to the catalytic unit of cellulose synthase and thus providing UDP-Glc directly for cellulose synthesis (Fujii et al., 2010). The *in vivo* roles of Arabidopsis SuSy have been the subject of much debate, involving evidence for and against the involvement of SuSy in cellulose synthesis (e.g., Barratt et al., 2009; Baroja-Fernández et al., 2012a,b; Smith et al., 2012). On the other hand, recent studies have shown that reducing the SuSy activity in stems of alfalfa and poplar by approx. 95 or 94% (by co-suppression and RNAi, respectively) caused no major growth alterations. This suggested that SuSy activity is not essential for providing UDP-Glc to cellulose synthase in those plants but appears to be involved in sucrose metabolism (Gerber et al., 2014; Samac et al., 2015). Besides producing UDP-Glc, SuSy was also suggested to have an important role in ADP-Glc synthesis, by using ADP instead of UDP, as one of its substrates. The produced ADP-Glc could then be used for starch biosynthesis (e.g., Baroja-Fernández et al., 2003, 2004, 2009; Bahaji et al., 2014; Baslam et al., 2017; but see also Fernandez et al., 2017). The roles of SuSy (and the controversies involved) were recently reviewed by Kleczkowski and Decker (2015).

### UDP-Sugar Interconversion Mechanisms

UDP-sugars formed *de novo* by the pyrophosphorylases can subsequently be converted to other nucleotide sugars by various secondary mechanisms (Figure 3), such as the nucleotide-sugar oxidative pathway by UDP-Glc dehydrogenase and UDP-GlcA decarboxylase, forming UDP-GlcA and UDP-Xyl, respectively. Products of this pathway can then be used as substrates for a number of epimerases to form their corresponding 4-epimers (such as UDP-Gal, UDP-GalA and UDP-Ara). In barley, it was shown that a C4-epimerase can interconvert UDP-GlcNAc and UDP-GalNAc (Zhang et al., 2006). UDP-Glc can also be used as a precursor for synthesis of UDP-sulfoquinovose or UDP-rhamnose. The previously mentioned UDP-sugars are pyranoses, but UDP-GlcA may be used to form UDP-apiose, which is in furanose form. UDP-Ara may also be converted into its furanose form by a mutase. No mechanisms for direct conversion between the pools of UDP-sugars and UDP-NAc-sugars have been reported (Bar-Peled and O’Neill, 2011). More comprehensive and in depth descriptions of the UDP-sugar interconversion mechanisms can be found in the studies of Bar-Peled and O’Neill (2011), Yin et al. (2011), and Kleczkowski and Decker (2015).

## Structural Determinants of Substrate Specificity of the Pyrophosphorylases

**Figure 4:**
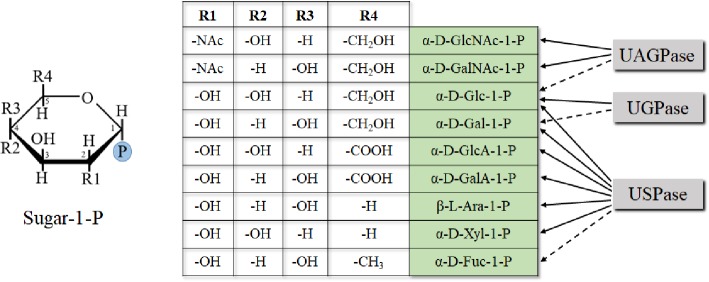
Chemical determinants of sugar-1-P (based on pyranose ring) specificity of UDP-sugar producing pyrophosphorylases. Structures of Fru-1-P and Fru-2-P (less specific substrates of UGPase) are based on furanose ring and are not shown. Dotted lines refer to less specific reactions.

Studies on enzyme specificity of the UDP-sugar metabolizing pyrophosphorylases have commonly been performed using a single enzyme to examine a range of sugar-1-phosphates and nucleoside triphosphates (Ritter et al., 1996; Litterer et al., 2006b; Kotake et al., 2007; Yang et al., 2010). The choice of tested substrates usually reflected their commercial availability at the time of the studies and what was already known about substrate specificity of a given enzyme. Recently, we have comprehensively tested substrate specificities of several UDP-sugar producing pyrophosphorylases, including barley and Arabidopsis (two isozymes) UGPases, Arabidopsis USPase and Arabidopsis UAGPase2, using as many as 11 different sugar-1-phosphates and 5 nucleoside triphosphates (Decker and Kleczkowski, 2017). Neither of the tested pyrophosphorylases appeared to favor purine substrates (ATP or GTP) nor the pyrimidine CTP, but all preferentially used UTP as nucleotide donor, and they differed in their specificity for sugar-1-P (Decker and Kleczkowski, 2017) (Figure 4). The specificities were mostly similar to those earlier reported for various UGPases, USPases, and UAGPases (reviewed in Kleczkowski et al., 2010, 2011a,b; Bar-Peled and O’Neill, 2011; Kleczkowski and Decker, 2015), but there were some new or rarely reported findings, for example, activity of UGPases with β-D-Fru-1-P and Fru-2-P (Km over 10 mM for barley UGPase) or activity of USPase with α-D-Fuc-1-P (Km of 3.4 mM) The roles of such reactions *in vivo* are mostly unclear. Fru-1-P has no known synthesis pathway; however, activated forms of Fru-2-P have been reported in plant tissues (Taniguchi, and Nakamura, 1972), and naturally occurring sugar bonds are more commonly activated on the anomeric carbon (Decker and Kleczkowski, 2017). Regarding UDP-α-D-Fuc in plants, there is no determined role either, and no changes in Fuc could be observed in cell wall or in leaf extracts in a strong USPase knock-down (Geserick and Tenhaken, 2013b). UDP-α-D-Fuc may perhaps have a role in secondary metabolism (Decker and Kleczkowski, 2017).

The sugar-1-phosphates used by the pyrophosphorylases cover a wide range of configurations/substituents on several positions of the sugar (Figure 4). Generally, enzyme affinities toward different substituents on C2, C4, and C5 of the pyranose ring of a sugar were crucial determinants of substrate specificity of a given pyrophosphorylase. The data obtained from the initial substrate-combination screens were used to construct a rudimentary quantitative structure-activity relationship model, which suggested a few additional substrate combinations to examine further, when substrates become commercially available (Decker and Kleczkowski, 2017). Comparisons of structural models of UDP-sugar binding to UGPase, USPase and UAGPase2 revealed similarities in UDP-moiety binding and differences in sugar binding, which may partially explain differences in substrate specificity of these proteins (Decker and Kleczkowski, 2017). These models provide a preliminary structural basis for rational manipulation of substrate specificities of the pyrophosphorylases. A similar rationale, based on comprehensive comparisons of several unrelated oxidoreductases, has allowed to successfully manipulate the specificity of cofactor used during the reaction (from NADP to NAD) (Cahn et al., 2017).

## Chemical Genetics to Study *in Vivo* Functions of the Pyrophosphorylases

Classical reverse genetics studies to elucidate *in vivo* functions of UDP-sugar metabolizing pyrophosphorylases have frequently been hampered by the fact that plant mutants with impaired expression of a given gene were defective in their reproductive abilities (e.g., Schnurr et al., 2006; Kotake et al., 2007; Meng et al., 2009b; Park et al., 2010; Geserick and Tenhaken, 2013a; Chen et al., 2014). The resulting male sterility or other fertility problems prevent obtaining homozygous mutants for those proteins and, to a large extent, preclude more detailed information on the *in vivo* importance of those proteins, in processes other than fertility-oriented. In addition, other genes coding for related proteins (e.g., USPase in an UGPase mutant) could possibly compensate for some aspects of a silenced UDP-sugar metabolizing pyrophosphorylase (Meng et al., 2009b). These problems call for more refined approaches to define physiological roles of the UDP-sugar metabolizing pyrophosphorylases. An example of such an alternative approach is so-called reverse chemical genetics, which postulates the use of specific inhibitors affecting one or more of the pyrophosphorylases *in vivo*.

In an attempt to provide a complement to the previous studies in which UDP-sugar producing pyrophosphorylases were interfered at gene or transcription level, we have recently identified several compounds which strongly inhibited the activity of the enzymes. The compounds were identified by screening the activities of heterologously expressed purified barley UGPase and *Leishmania major* USPase against chemical library of small molecular mass compounds (Decker et al., 2017). We used a novel bioluminescence-based assay system (Decker et al., 2014) that gave a quantitative measurement of UGPase and USPase activities, which could be detected down to a pmol/min level. The identified compounds inhibited both UGPase and USPase activities, with IC_50_ values of ca. 50 μM or lower. Subsequent assays with Arabidopsis USPase revealed that it was similarly affected by the inhibitors such as Leishmania USPase (Decker et al., 2017). The strong inhibitory effects of several compounds on distinct pyrophosphorylases from barley (UGPase) as well as Arabidopsis and Leishmania (USPase) suggest common structural determinants at, or close to, their active sites, consistent with earlier analyses (Geisler et al., 2004; Kleczkowski et al. 2011b). Hit expansion on one of the inhibitors, a salicylimide-derived compound (cmpd #6), yielded several analogs with a range of activities on both UGPase and USPase (Decker et al., 2017, 2018). Two of the most efficient inhibitors, cmpd #6D and #6D2, were then used for detailed kinetic studies of the pyrophosphorylases. In a separate study (Decker and Kleczkowski, 2017), cmpd #6D was also found to inhibit Arabidopsis UAGPase2, again suggesting that the inhibitor may target a common feature of the pyrophosphorylases.

Both cmpd #6D and #6D2 acted as uncompetitive inhibitors of purified UGPase and USPase (Decker et al., 2017). This implies that their effects cannot be overcome by increased substrate concentration, and thus, they are very suitable for *in vivo* experiments (Kleczkowski, 1994b). The compounds were found as potent inhibitors of Arabidopsis cell culture growth and Arabidopsis pollen germination (Decker et al., 2017). In the latter case, addition of UDP-Glc or UDP-Gal relieved the inhibitory effect, suggesting that the inhibitors targeted UDP-sugar formation. Overall, the results suggested that inhibitors may represent useful tools to study *in vivo* roles of the UDP-sugar metabolizing pyrophosphorylases and may serve as a complement to the genetic approaches. Further hit expansion on cmpd #6 analogs and, perhaps, on other inhibitors identified during the chemical library screening may yield compounds, which will be even more potent against the pyrophosphorylases and could be useful for *in vivo* studies. In such a search, priority should be given to inhibitors that *specifically* affect a given pyrophosphorylase activity (Decker et al. 2017, 2018; Decker and Kleczkowski, 2017).

Screening for, or designing, specific inhibitors of various pyrophosphorylases has been the subject of several recent studies. The pyrophosphorylases’ general role as providers of direct precursors for formation of essential glycosylated end-products makes them attractive targets for drug and pharmaceutical industries to develop efficient compounds which can selectively target pathogens. For instance, several novel inhibitors were identified for GDP-Man pyrophosphorylase from two different species of Leishmania (Mao et al., 2017). The inhibitors were rationally designed from docking analyses, and some of them exerted stronger effects on leishmanial GDP-Man pyrophosphorylases than on corresponding human enzyme, suggesting a pharmaceutical potential. Also recently, using a novel rational approach (based on dynamics of the enzymatic reaction cycle), a non-conserved allosteric site, distant from the active site and suitable for inhibitor binding, was identified for *Leishmania major* UGPase (Cramer et al., 2018). The site was specific for the pathogen enzyme and was not present in human UGPase. A molecular scaffold, developed for allosteric targeting of the Leishmania UGPase, led to the identification of murrayamine-I (a carbazole alkaloid) as selective inhibitor of the enzyme. Such a rational targeting of non-conserved allosteric sites of a protein not only opens up the possibilities for new therapies and biotechnologies (Cramer et al., 2018) but also can dramatically help in studies on physiological roles of a given protein.

## Perspectives

Although UDP-sugar metabolizing pyrophosphorylases have been studied for many years (especially UGPase), there is still need for further studies. Because of the difficulties in producing viable knockouts, due to reproductive impairment of the resulting mutants (Park et al., 2010; Geserick and Tenhaken, 2013a,b; Chen et al., 2014), we still do not know exactly what are their *in vivo* roles, beyond their involvement in reproductive processes. As the family of enzymes responsible for *de novo* formation of UDP-sugars, they are most likely linked to biomass production (e.g., cell wall biosynthesis). However, such a role could only be demonstrated for some transgenic plants with overexpressed plant UGPase (Wang et al., 2011) or UAGPase (Jiang et al., 2015). Chemical genetics may provide alternative to the mutant approaches (see below).

Additional basic studies may reveal the fine details on how plants control their UDP-sugar metabolizing activities by transcriptional, translational, and post-translational control, but also at the level of enzyme regulation by metabolic effectors. This should contribute to a more comprehensive understanding as to how UGPase, USPase, and UAGPase, together with the UDP-sugar-interconverting enzymes, function *in planta* to serve the needs of different tissues, developmental stages, and in response to different conditions. For instance, very little is known about the significance of reported post-translational modifications (phosphorylation and redox regulation) of plant UGPase (Soares et al., 2014). Also, more studies are needed to analyze further the mechanism of (de)oligomerization of plant UGPase (Figure 2A).

Among plant UDP-sugar metabolizing pyrophosphorylases, only Arabidopsis UGPase1 and sugarcane UGPase have been crystallized and had their structures resolved (McCoy et al., 2007; Cotrim et al., 2018). Structures of plant USPase and UAGPase can only be modeled on corresponding proteins from non-plant eukaryotes, that is, *Leishmania major* in the case of USPase (Dickmanns et al., 2011), and mammals and yeast—for UAGPase (Peneff et al., 2001; Maruyama et al., 2007). Given the differences in substrate specificity between different UDP-sugar metabolizing pyrophosphorylases (Kleczkowski and Decker, 2015; Decker and Kleczkowski, 2017), such resolved and predicted models may aid in understanding which amino acids determine substrate specificity**.**

### Origin of “Unusual” UDP-Sugars

Plant extracts contain about 30 nucleotide sugars, most of them UDP-sugars, and the origin of some of them is unknown. As an example, synthesis of rhamnogalacturonan-II, a pectic polymer present in cell walls, requires the presence of at least 12 different nucleotide sugars. Also, some of the cell wall glycans are composed of sugars or sugar derivatives for which the corresponding nucleotide sugars are unknown, for example, 3-deoxy-D-*lyxo*-2-heptulosaric acid (Dha) or aceric acid (Bar-Peled and O’Neill, 2011). One of the ways to address this is to continue systematic screening for substrate specificity of several NDP-sugar pyrophosphorylases, not only those covered in this review, using as wide range of sugar-1-phosphates and NTPs as possible. Also, NDP-sugar interconverting enzymes (e.g., GDP-Man epimerase) as well as SuSy and phosphorylases capable of activating a sugar with NDP as substrate (e.g., ADP-Glc phosphorylase) (Kleczkowski and Decker, 2015) could be used. In case of SuSy, one may use different NDPs as substrates as well as synthetic di-sugars, in addition to sucrose (Sauerzapfe et al., 2008). Such a broad search could possibly reveal the mechanisms/origin of those nucleotide sugars that are not produced by either UGPase/USPase/UAGPase activity but were earlier reported to be present in plant preparations (Bar-Peled and O’Neill, 2011; Kleczkowski and Decker, 2015). Importantly, one could repeat the same type of substrate-specificity screens for the same enzymes, but purified from plant extracts or overexpressed/purified using a eukaryotic system (e.g., yeast) rather than overexpressed/purified in prokaryotic systems. This would allow to determine whether post-translational modifications have any effect on substrate specificity of a given NDP-sugar producing enzyme. A similar comparative study found striking differences in substrate specificity for potato SuSy1 (Sauerzapfe et al., 2008), whereas there were no apparent differences for pea USPase purified from plant extracts or after overproduction in bacteria (Kotake et al., 2004).

### Directed Evolution and Structure-Guided Approaches to Change Substrate Specificity

Eukaryotic UDP-sugar producing pyrophosphorylases, although differing substantially in their amino acid sequences (only ca. 10–20% homology), share common structural blueprint and at least some details of their active sites (Geisler et al., 2004; Kleczkowski et al., 2011b). Structural similarities at or nearby substrate-binding domains are presumably also the case for bacterial nucleotide-sugar producing pyrophosphorylases, even though those proteins frequently share less than 10% identity with their plant counterparts (Kleczkowski et al., 2004). In one study, a mild random mutagenesis of a bacterial AGPase resulted in the production of novel proteins which had their substrate specificity changed to that of UGPase and UAGPase (Sohn et al., 2006). Thus, the nucleotide-sugar producing enzymes do have the capacity for changing their substrate specificity after few mutations. Recent studies by Ebrecht et al. (2017), also using bacterial AGPase, have suggested a role for allosteric regulation of certain enzymes as an evolutionary mechanism for the selection of specific substrates.

It is yet unknown whether the “directed evolution” approach would work with plant pyrophosphorylases. However, even if it does result in a change of specificity, the effects of the mutation(s) may modulate affinity of substrate binding and/or catalysis rate. A frequent problem in such studies is that substrate specificity is nearly always determined not by one but by several amino acids, and changing specificity may require multiple simultaneous mutations, rapidly expanding the number of possibilities to explore. This calls for a more comprehensive heuristic approach, taking into account previous studies focused on structure/function relationship for pyrophosphorylases. This could leverage the diversity of substrates effectively taken by active sites and limit the scope to an experimentally tractable scale. A similar structure-guided semi-rational strategy has recently allowed to change the cofactor specificity (from NADP to NAD-preferring) of several structurally diverse oxidoreductases (Cahn et al., 2017).

Future studies of UGPase, USPase, and UAGPase should also aim at understanding how the enzyme/substrate interactions are changing during the catalytic cycle, similarly to what was described for *Leishmania* UGPase (Führing et al., 2013a), and which was crucial to identify allosteric sites, distant from the active site (Cramer et al., 2018). However, until resolved crystal structures are available for plant USPase and UAGPase, we may only rely on useful, but not perfect, homology models in further elucidating the substrate/product cycle for those proteins.

### Inhibitor Approaches

One of the most important questions in chemical genetics is whether the *in vivo* effects of a given inhibitor indeed reflect its *in vitro* effects on a given purified protein/enzyme or are the result of inhibition of multiple targets. Since plant cells contain thousands of distinct proteins, one should limit the possibility of multiple unspecific targets. One of the ways to address this may involve search for allosteric non-conserved regions of a pyrophosphorylase, similar to the approach by Cramer et al. (2018) (see above). This approach, validated for Leishmania UGPase, may result in the identification of unique non-conserved allosteric sites, allowing to design specific inhibitors, targeting not only a specific class of pyrophosphorylases (e.g., UGPase, but not UAGPase or USPase) but also allowing to distinguish between representatives of a given class (e.g., Arabidopsis UGPase-A1 vs. Arabidopsis UGPase-A2). Compounds that are tailor-designed to bind to a given non-conserved allosteric site have higher probability to act on unique targets than compounds identified by a general chemical library screening. In this way, chemical genetics may truly rival classical genetics (mutants), providing tools for *specific* interference.

### UDP-Glc as Signaling Molecule?

In animals, UDP-Glc has a well-defined role of an extracellular signal, which is sensed by G protein-linked receptors, and activating several transduction pathways (Freeman et al., 2001; Harden et al., 2010; Lazarowski and Harden, 2015). This has never been shown for plants. However, there is some evidence that reduced content of UDP-Glc results in abnormal growth, which could be rescued by external provision of UDP-Glc, whereas other studies pointed out links between UDP-Glc content and sensitivity to a pathogen-induced cell death (Chivasa et al., 2013; Wang et al., 2015; Xiao et al., 2018; Janse van Rensburg and Van den Ende, 2018). As the UTP-dependent pyrophosphorylases are the primary *de novo* producers of UDP-Glc, they would certainly be important players in UDP-Glc signaling. Indeed, UGPase1 and UAGPase2 were identified as novel regulators of PCD in Arabidopsis and rice, respectively (Chivasa et al., 2013; Xiao et al., 2018). Further studies, as outlined by Janse van Rensburg and Van den Ende (2018), are required on putative signaling role(s) by UDP-Glc and, possibly, other nucleotide sugars in plants.

## Author Contributions

DD wrote the original draft of the manuscript, LK edited the draft and prepared the final version. Both authors have made a substantial, direct and intellectual contribution to the work, and approved it for publication.

### Conflict of Interest Statement

The authors declare that the research was conducted in the absence of any commercial or financial relationships that could be construed as a potential conflict of interest.

## Footnotes

^1^ http://www.cazy.org/

^2^ http://plantcyc.org/
